# Ribo-Pop: simple, cost-effective, and widely applicable ribosomal RNA depletion

**DOI:** 10.1261/rna.076562.120

**Published:** 2020-11

**Authors:** Mary Kay Thompson, Maria Kiourlappou, Ilan Davis

**Affiliations:** Department of Biochemistry, University of Oxford, Oxford, OX1 3QU, United Kingdom

**Keywords:** *Drosophila*, RNA sequencing, ribosomal RNA depletion

## Abstract

The measurement of RNA abundance derived from massively parallel sequencing experiments is an essential technique. Methods that reduce ribosomal RNA levels are usually required prior to sequencing library construction because ribosomal RNA typically comprises the vast majority of a total RNA sample. For some experiments, ribosomal RNA depletion is favored over poly(A) selection because it offers a more inclusive representation of the transcriptome. However, methods to deplete ribosomal RNA are generally proprietary, complex, inefficient, applicable to only specific species, or compatible with only a narrow range of RNA input levels. Here, we describe Ribo-Pop (ribosomal RNA depletion for popular use), a simple workflow and antisense oligo design strategy that we demonstrate works over a wide input range and can be easily adapted to any organism with a sequenced genome. We provide a computational pipeline for probe selection, a streamlined 20-min protocol, and ready-to-use oligo sequences for several organisms. We anticipate that our simple and generalizable “open source” design strategy would enable virtually any laboratory to pursue full transcriptome sequencing in their organism of interest with minimal time and resource investment.

## INTRODUCTION

Researchers across the biological sciences use RNA sequencing to generate novel insights or test hypotheses. However, in most organisms, ribosomal RNA comprises the majority of the RNA sample, making it economically impractical to analyze sequences of interest such as protein-coding mRNAs and other nonribosomal transcripts at substantial coverage. Therefore, efficient sequencing requires either capture of the RNAs of interest or depletion of ribosomal RNA.

The most widely used capture method is poly(A) selection, in which the polyadenylated RNAs are purified by hybridization to oligo(dT). Ribosomal RNA, which is not polyadenylated, is greatly reduced in poly(A)-selected sequencing libraries. However, in recent years, interest in the nonpolyadenylated transcriptome has grown considerably. Studies which seek to characterize preadenylation steps in RNA processing such as nascent transcription and splicing cannot use poly(A) selection (Ameur et al. 2011; Khodor et al. 2011). Furthermore, some transcription events produce RNAs without poly(A) tails. Many noncanonical transcripts, including some classes of enhancer RNAs and anti-sense transcripts, are thought to be unadenylated (Katayama et al. 2005; Kim et al. 2010). Moreover, many gene products, such as certain long noncoding RNAs and histone mRNAs, are not polyadenylated, and poly(A) selection does not effectively recover these species (Yang et al. 2011; Livyatan et al. 2013). Poly(A) selection can also distort abundance measurements of polyadenylated RNAs. RNAs with short poly(A) tails are not captured efficiently by poly(A) selection, and thus use of poly(A) selection can vastly distort gene expression measurements in many contexts (Weinberg et al. 2016). In certain exacting experiments, such as measurement of translation or RNA turnover, measurement of polyadenylated RNA rather than the body of the RNA can lead to qualitatively different conclusions (Presnyak et al. 2015; Weinberg et al. 2016).

To address these shortcomings, removal of ribosomal RNA, rather than selection of a poly(A) tail, has been introduced in many experimental workflows. Unfortunately, removal of ribosomal RNA is more complicated than poly(A) selection because there is no universal reagent equivalent to oligo(dT) available. Although many protocols and commercial products have been developed to deplete ribosomal RNA or its cDNA derivatives, most of these solutions suffer from various pitfalls that prevent their widespread use. First, each reagent is only applicable to a specific organism or family because hybridization approaches require a high degree of sequence complementarity. For example, as of writing, several commercial kits that deplete rRNA from mammalian samples are available, but none are available for *Drosophila*. Because of the proprietary nature of these kits, researchers working in other organisms cannot benefit from any knowledge used to produce these probe sets in order to design probe sets for their organism of interest. Secondly, even commercial kits are unsuitable for certain types of experiments in their target organism(s). Some of them have narrow RNA input ranges or are not made available as a separate component outside of a sequencing library construction kit, making them difficult to use for novel or exploratory experiments that may deviate from a traditional workflow. Finally, the cost of commercial rRNA depletion reagents is prohibitive for some research groups and prevents their wider adoption.

In summary, we argue that the current lack of versatile and cost-effective rRNA subtraction reagents severely restricts exploration in many areas of RNA biology. Here, we address this problem by developing an “open source” rRNA depletion solution that is simple to use, cost-effective, and can be easily adapted to any organism of interest. We validated the use of our rRNA subtraction method using RNA purified from *Drosophila* and *S. cerevisiae*, and we created an automated probe design pipeline to enable selection of new probe sets. We named our method Ribo-Pop (ribosomal RNA depletion for popular use) because it democratizes rRNA depletion, enabling researchers working with any organism or workflow to deplete ribosomal RNA in an efficient and cost-effective manner.

## RESULTS

**FIGURE 1.**
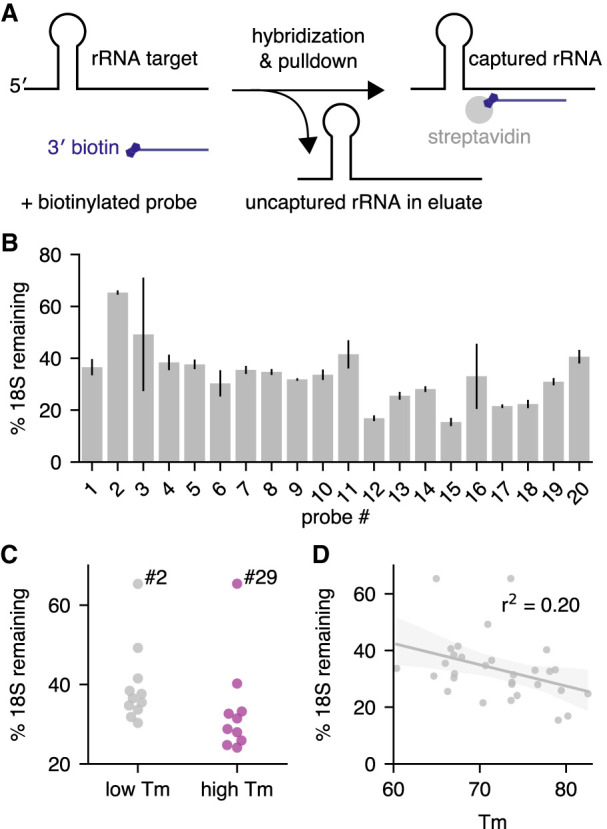
Short antisense oligos effectively deplete ribosomal RNA. Oligonucleotides targeting the *Drosophila* small rRNA (18S) were individually tested for their ability to deplete the *18S* transcript from larval total RNA. The percent of remaining 18S was quantified by qPCR. Values are derived from *18S* normalized to *Act5c*, in turn normalized to a nondepleted sample (no probe control). Values are the averages of three replicates of the depletion experiment, each using a different sample of larval RNA. (*A*) Outline of the single-probe depletion assay. A 3′ biotinylated probe targeting a specific site in the 18S is added to total RNA and subjected to hybridization. The target is captured with streptavidin beads and the remaining target is measured from the supernatant. (*B*) The percent of 18S rRNA remaining for all tested oligos of size 26–32 nt, arranged 5′ to 3′ by target site. Error bars are standard deviation between separate hybridization experiments, each performed with a different RNA sample. (*C*) Performance comparison between the initial set of low *T*_m_ probes (probes #1–#11, *T*_m_ < 72°C) targeting the left side of the 18S transcript and the second set of high *T*_m_ probes (probes #21–30, *T*_m_ > 72°C). Two-sided *t*-test *P* = 0.12. Probe #2 and probe #29 are outliers, possibly for structural reasons (see Fig. 2). (*D*) Correlation between the predicted *T*_m_ of the probe/target hybrid and the percent of remaining target for the 30 ∼30mer probes tested in the single-probe depletion assay (*P* = 0.01, Spearman's correlation).

In an effort to develop high-efficiency ribosomal RNA removal, we decided to test short, biotinylated oligonucleotides for their ability to deplete ribosomal RNA by hybridization and subsequent removal of the rRNA/probe hybrids via pulldown with streptavidin-coupled beads. First, we tested how much rRNA depletion is achieved from a single probe/target interaction, in order to determine the minimum number of high affinity probes required to purify unfragmented rRNA. We designed and biotinylated 20 short ∼30mer probes (26–32 nt) targeting the small 18S rRNA transcript using parameters developed for microarray probe design and used in smFISH experiments (Supplemental Fig. S1; Tsanov et al. 2016; Gaspar et al. 2017). Surprisingly, all probes were able to deplete rRNA to some degree on their own, compared to a control sample containing no probe that was processed in parallel. Each probe was tested individually for its ability to deplete the small 18S rRNA transcript from *Drosophila* total RNA at an estimated fivefold molar excess in a single-probe depletion assay (Fig. 1A). In the single-probe depletion assay, probe and total RNA were mixed in a standard hybridization buffer (2X SSC, 0.01% Tween-20) and denatured at 70°C for 5 min. After annealing for 10 min at room temperature, the biotinylated probes were captured with streptavidin beads and the remaining rRNA was measured from the supernatant. The average depletion was threefold, corresponding to 33% of 18S remaining compared to the no probe control sample (Fig. 1B; Supplemental Table S1). These results show that many different probes are effective, despite the highly structured nature of the ribosomal RNA molecule.

We chose to use short ∼30mer probes for several practical and theoretical reasons. Short probes are inexpensive to manufacture at high purity, and they have lower potential for hairpin formation, unwanted dimerization, and off-target binding (Chou 2004). However, some studies of microarray probe design suggest that longer probes perform better in that application (Chou 2004). We therefore tested whether longer probes designed using our sequence composition criteria would be more efficient at removing rRNA. We found little evidence that longer probes of 46–52 nt removed more rRNA than the short probes (Supplemental Fig. S2A). We hypothesize that the additional nucleotides present in the longer probes make the propensity for self-structure greater and offset the theoretical gain in target affinity. Therefore, we decided to continue using small probes and turned our focus to optimization of probe selection within the 30 nt size range.

We examined our depletion data in order to discover probe properties favorable for ribosomal RNA depletion. Although all probes were able to achieve some degree of depletion, we observed large differences between the best- and worst-performing probes (Fig. 1B). To determine whether thermodynamic properties might explain these differences, we analyzed their relationship with probe efficacy. The correlation between the predicted ΔG for hairpin and homodimer formation of each probe and target depletion were not significant (Supplemental Fig. S2B,C). In contrast, the predicted *T*_m_ of each probe bound to its target was convincingly correlated with target depletion, with higher *T*_m_ probes achieving greater depletion on average (*r*^2^ = 0.17, *P* = 0.07, Spearman's correlation) (Supplemental Fig. S2D).

To test whether probes with *T*_m_s that are relatively high (>72°C) compared to the surrounding sequence area would perform better, we designed probes corresponding to *T*_m_ peaks in the first 60% of the 18S target region and tested them for their ability to deplete 18S rRNA. We observed that higher *T*_m_ probes did perform better than lower *T*_m_ probes on average (3.3-fold vs. 2.7-fold median depletion), although the comparison was not statistically significant (*P* = 0.12) (Fig. 1C; Supplemental Fig. S2E). Nevertheless, when we include all our tested ∼30mer probes in the analysis, *T*_m_ and probe performance are significantly correlated (Fig. 1D, *r*^2^ = 0.20, *P* = 0.01, Spearman's correlation). Given this striking correlation, we decided to set high *T*_m_ as one of our probe selection criteria.

Because we planned to select probes with *T*_m_s higher than the denaturation temperature (70°C), we reasoned that annealing would likely begin during the denaturation step, and that the annealing step of the protocol could be eliminated without affecting performance. We tested three different probes with and without the annealing step (Supplemental Fig. S3). Removing the annealing step completely by adding the denatured RNA directly to beads that had been preheated to 50°C increased the remaining 18S by an average of 11% across three probes tested, which ranged in *T*_m_ from 65°C to 80°C (*P* = 0.04). Given the small size of the effect, we decided to eliminate the 10 min annealing step from our further experiments and moved quickly from the denaturation step to streptavidin binding, allowing the hybridization reactions to rest only briefly at room temperature (∼1 min) before proceeding to bead capture. Thus, simply by choosing high *T*_m_ probes, we were able to create a rapid and robust rRNA depletion strategy.

**FIGURE 2.**
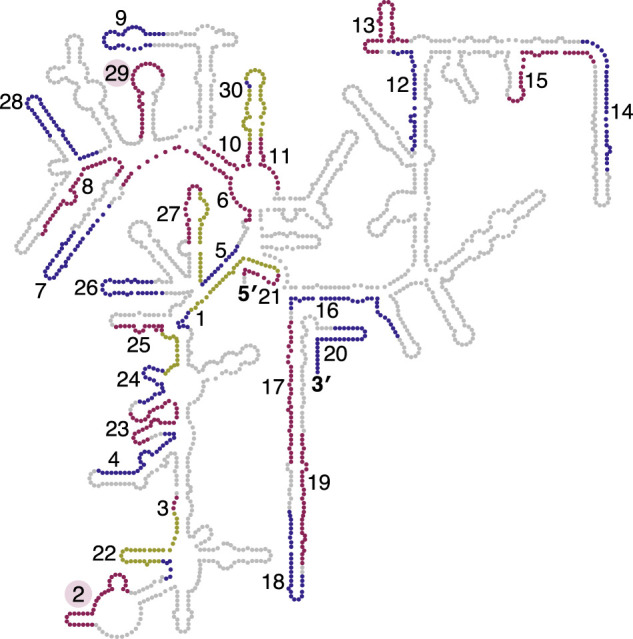
Most 18S structural features are accessible to probe targeting. The target sites of tested probes in the *Drosophila* 18S rRNA are shown. The secondary structure representation is based on data from the 3D structure and constructed using the RiboVision program (Anger et al. 2013; Bernier et al. 2014). Some probe target sites are overlapping because we designed a second set of high *T*_m_ probes against the 5′ side of the 18S (probes #21–30, see Fig. 1). To distinguish between the individual target sites, we have alternated the colors between red and blue, with the overlapping portions of probe target sites shown in yellow. The labels for the target sites of probe #2 and #29 are highlighted in red.

We then asked whether local or global structure of the target rRNA molecule affects probe performance. We examined the target sites of the 18S probes in the structure of the small ribosomal subunit (Fig. 2; Anger et al. 2013; Bernier et al. 2014). Interestingly, the target sites of successful probes are distributed across the structure with no obvious propensity for single-stranded regions. In fact, many successful probes overlap hairpins that would be expected to require unfolding for the probe to bind. The two worst-performing probes (probe #2 and probe #29, Fig. 1C) overlap with regions of ribosomal RNA expansion segments ES3 and ES6 that are known to interact by base-pairing in the 3D structure, forming a helix (Supplemental Fig. S4; Alkemar 2003; Armache et al. 2010). This ES3/ES6 helix comprises eight consecutive base pairs, but it seems unlikely that this fact alone can explain the poor performance of these probes. Although probe #29 directly interacts with residues involved in the base-pairing, probe #2 interacts with residues adjacent to the helix that are not directly involved in base-pairing. Furthermore, several effective probes (#4, #8, #14, #18, #20, and #28) target regions containing at least eight consecutive base pairs. We postulate that the ES3/E6 long-range tertiary interaction is not completely unfolded after denaturation at 70°C. In some RNA structures, long-range tertiary interactions form cooperatively at an early stage of the folding pathway and may be more stable than expected (Behrouzi et al. 2012; Koculi et al. 2012). If probe #2 and probe #29 are excluded from the high and low *T*_m_ probe sets based on their proximity to the ES3/ES6 helix, then the difference between high and low *T*_m_ performance becomes statistically significant (*P* = 0.005, Fig. 1C). Taken together, our survey of 18S target sites suggests that successful probes can be chosen by picking probes with predicted *T*_m_ above the denaturation temperature and avoiding probes that are likely to contact a high affinity long-range interaction in the rRNA molecule.

Using our selection criteria of short, high *T*_m_ probes outside of deeply structured regions, we chose a set of 15 probes covering the small and large ribosomal RNA transcripts (five targeting the 18S, five targeting the 5′ fragment of the 28S [28S-L], and five targeting the 3′ fragment of the 28S [28S-R]), spaced evenly along the transcripts to yield a probe density of approximately 1 probe/400 nt. We did not include probes for the smaller transcripts (5S and 5.8S) because we observed that they are not well captured by most RNA-seq protocols and do not contribute substantially to the total rRNA contamination. For the 18S probes, we selected five probes from those tested in Figure 1 (probes #12, 18, 21, 24, and 28). For the large subunit 28S fragments, we chose evenly spaced probes conforming to our sequence composition constraints and falling within the top 5% of *T*_m_ for potential probes. We also screened probes for potential matches against processed and unprocessed transcripts, using an alignment score cutoff that excludes probes matching more than 15 consecutive base pairs to nontargets (Kane 2000). We note that this filter is more stringent than many probe filtering pipelines because we included screening against genomic regions falling within annotated gene boundaries, that is, intronic sequences found in unprocessed transcripts. Thus, our probe design should allow us to deplete the major ribosomal RNA transcripts with minimal effects on both processed and unprocessed RNA quantification.

**FIGURE 3.**
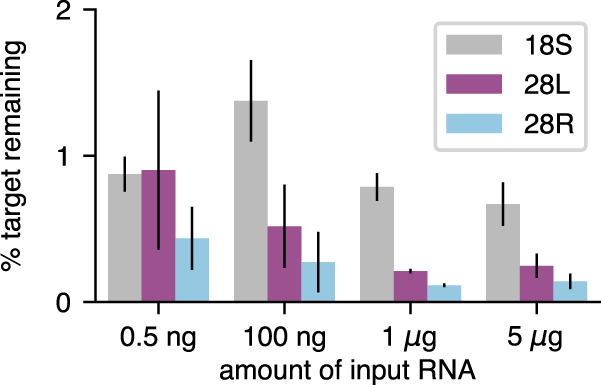
Depletion of rRNA performs well across a range of total RNA input levels. The percent of 18S, 28S-L (28L), and 28S-R (28R) targets remaining after Ribo-Pop starting from various levels of input RNA are shown. qPCR values for each target were normalized to the *Act5c* level of the same sample and then normalized to an input sample that was not subjected to Ribo-Pop treatment. See Materials and Methods and Supplemental Protocol for a detailed protocol. Error bars are the standard deviation between three independent experiments, each using a different RNA sample. Sequencing data from the 5 µg samples are presented in Figure 4.

We first tested the efficacy and input range of our 15-probe pool using *Drosophila* larval total RNA. For larger RNA input amounts (1–5 µg), we successfully used an estimated fivefold molar excess of probe mix to rRNA (40 pmol probe mix per µg of input RNA) to deplete targets to <1% of their input levels (Fig. 3). Our optimization experiments demonstrated that a higher probe excess was needed to achieve similar depletion levels when lower input amounts were used (data not shown), consistent with the expectation that hybridization should be concentration dependent. We therefore used an estimated 20-fold molar excess to deplete rRNA from 100 ng of input RNA (Fig. 3). We were able to deplete rRNA from as little as 0.5 ng input using the 100 ng reaction conditions (probe mix at 4000-fold molar excess relative to 0.5 ng input RNA, Fig. 3). As the typical cell contains 10–20 pg of RNA (Ogura et al. 1998), 0.5 ng represents the amount of RNA that may be obtained from only 25–50 cells, which is comparable to a rare cell type experiment. Therefore, we have shown Ribo-Pop is effective and applicable over at least a 10,000-fold range.

**FIGURE 4.**
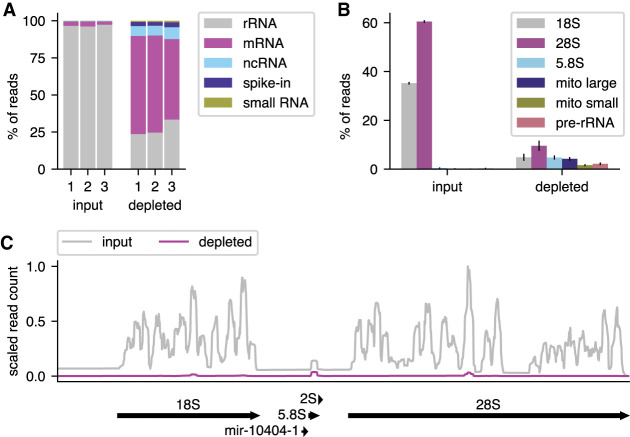
Ribo-Pop drastically increases effective sequencing depth. The effectiveness of Ribo-Pop depletion was assessed by RNA sequencing of input and depleted libraries prepared from *Drosophila* larval RNA. (*A*) The percent of reads assigned to each transcript class (biotype), is plotted for each of the three replicates of the input and depleted RNA samples. The data were obtained from three independent RNA samples in which each sample was used to construct one input library and one depleted library. (*B*) The percent of reads assigned to each type of ribosomal RNA is shown for the input and depleted samples. Error bars represent standard deviation between the three replicates. (*C*) RNA-seq coverage of a representative pre-rRNA locus (FBgn0267507) and its cleavage products in input and depleted samples. Reads in each library were scaled by the synthetic spike-in counts to allow direct comparisons between libraries. The average of three replicates for each sample type is plotted.

It was important to know what improvement in effective sequencing depth could be achieved by applying Ribo-Pop to a typical RNA sequencing experiment. We subjected 5 µg of *Drosophila* total larval RNA to Ribo-Pop depletion and prepared sequencing libraries from the remaining RNA. Nondepleted RNA-seq libraries (“input”) were prepared in parallel. After sequencing, we mapped the reads to all annotated transcripts and counted the sum of reads counted per transcript class (Fig. 4A). Ribo-Pop depletion decreased the percentage of reads assigned to rRNA transcripts from an average of 97% of reads in the input libraries to an average of 27% of reads in the depleted libraries. Concurrently, depletion greatly increased coverage on non-rRNA transcripts. Protein-coding transcripts increased from 2.7% to 61.9% of reads and ncRNAs increased from 0.4% to 7.1% of reads. We also added synthetic spike-in RNAs to the input samples before depletion, and coverage on these spike-ins increased from 0.1% to 2.8% after depletion. Hence, Ribo-Pop depletion increased the effective depth of the sequencing experiment more than 20-fold.

We then examined the remaining rRNA contamination in our depleted libraries. The remaining 27% of rRNA contamination is comprised of 9.6% 28S, 4.8% 18S, 4.2% mitochondrial large rRNA, 4.8% 5.8S rRNA, 2.2% pre-rRNA, 1.6% mitochondrial small rRNA, and 0.04% 5S (Fig. 4B). Thus Ribo-Pop depleted the targeted 18S and 28S RNAs down to levels similar to the minor rRNA contaminants (mitochondrial rRNAs, pre-rRNA, 5S and 5.8S), which together make up around 1% of reads in the input libraries. RNA depletion occurs relatively evenly across the targeted rRNA transcripts (Fig. 4C), suggesting that the remaining contamination is widely distributed. It is important to note that the observed increase in the percent of reads assigned to nontargeted RNAs can be explained by the increased sequence space available after depletion of the 18S and 28S transcripts. Likewise, examining the percent of target RNAs remaining is not an accurate measure of their fold change upon depletion. The actual fold change can be estimated by normalizing read counts to synthetic spike-in RNAs that were added before the depletion step. Using this normalization method, we find that the 18S and 28S decrease to approximately 0.5% and 0.6% of their input RNA levels, respectively, in agreement with our qPCR results. Hence, Ribo-Pop greatly depletes its targeted rRNA transcripts.

**FIGURE 5.**
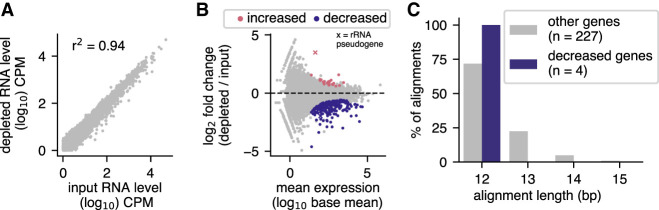
Ribo-Pop has minimal effects on expression measurements of non-rRNA genes. RNA-seq libraries prepared after Ribo-Pop depletion were compared to libraries made from nondepleted input RNA. To allow sufficient coverage for analysis, libraries were prepared using a poly(A)-primed method (QuantSeq). (*A*) Scatterplot and the Pearson's correlation between input and depleted RNA levels for mRNAs and ncRNAs. The RNA levels are the averages of three replicates for each condition, filtered to include only genes with an average of at least 1 count per million (CPM). Each replicate was prepared from a different RNA sample and corresponds to the same material used for the non-poly(A)-primed libraries analyzed in Figure 4. (*B*) MA plot for the comparison of Ribo-Pop depleted versus input libraries analyzed with DESeq2 (Love et al. 2014). Genes with an adjusted *P*-value <0.01 for differential expression are highlighted in pink if increased upon depletion or blue if decreased upon depletion. The two rRNA pseduogenes that are among the changing genes are indicated. (*C*) Stretches of complementarity between the Ribo-Pop probes and nontarget RNAs were identified by aligning the probe target sites to the transcriptome with BLAST (Altschul et al. 1990). Alignments are classified as overlapping a gene decreased upon Ribo-Pop depletion (decreased) or overlapping a gene not decreased upon depletion (other). Only alignments with 100% identity are displayed.

To test the specificity of our probe pool, we compared poly(A)-primed sequencing from depleted samples to poly(A)-primed sequencing of input samples. We chose to use poly(A)-primed data for this analysis because the low depth of sequencing in nonpoly(A)-primed, nondepleted input samples precludes quantification of most genes. We found that Ribo-Pop depletion had little effect on our gene quantification. The correlation between input and depleted RNA levels for mRNAs and ncRNAs was high (Pearson *r*^2^ = 0.94, Fig. 5A). We performed differential expression analysis and found that at an adjusted *P*-value cutoff of 0.01, 23 non-rRNA genes were called as increased by depletion and 208 non-rRNA genes were called as decreased, with a median fold-change of 1.9- and 2.2-fold, respectively (out of a total of 7,323 non-rRNA genes for which *P*-values could be calculated, Fig. 5B). If an adjusted *P*-value cutoff of 0.05 were used, 82 genes would be called as increased and 359 genes called as decreased with a median fold change of 1.8- and 1.9-fold, respectively. The decreased genes do not have more sequence complementarity to the probes or the targeted rRNA transcripts than all other genes, suggesting that our probe specificity filtering approach was successful (Fig. 5C; Supplemental Fig. S5A,B). The lack of substantial complementarity between the observed decreased genes and both the probes and the rRNA transcripts themselves suggests that Ribo-Pop does not cause codepletion of these RNAs by off-target hybridization. The RNA pools before and after depletion differ greatly in concentration and composition. It is possible that these differences cause over or under recovery of some specific RNAs during library preparation that we are detecting here. Overall, depletion does not substantially bias gene quantification.

**FIGURE 6.**
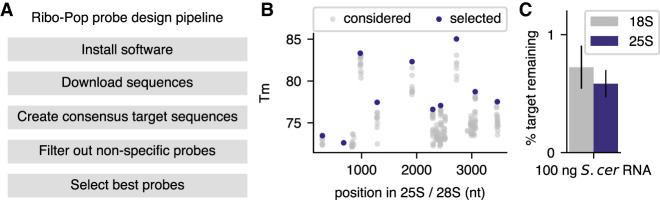
The Ribo-Pop probe design pipeline can be applied to other organisms. (*A*) The Ribo-Pop design pipeline. After software installation, the remaining steps can be automatically deployed. (*B*) An example output figure of the Ribo-Pop design pipeline after running the pipeline on the major rRNA transcripts of *S. cerevisiae* and *S. pombe* yeasts. The probe selection process for the large rRNA transcript is shown. Gray dots indicate possible probe placement sites based on conservation and sequence composition. Blue dots indicate the selected probes sites based on a desired probe set size of 10. (*C*) The Ribo-Pop probe set designed to target *S. cerevisiae* and *S. pombe* was applied to 100 ng of total RNA from *S. cerevisiae*. The averages of three experiments using different RNA samples are shown. Error bars are standard deviation.

Finally, our goal in developing Ribo-Pop was not merely to create an rRNA removal protocol for ourselves, but to provide a general solution for any researcher to apply to their organism(s) of interest. To this end we wrote a fully automated pipeline for probe selection, which includes selection of evenly spaced, high *T*_m_ probes and specificity checking (Fig. 6A). The pipeline is implemented in Snakemake (Koster and Rahmann 2012), which makes the entire probe design process deployable with a single command. We also predesigned probe sets for some common model organisms, where possible generating a single probe set to target multiple organisms (Supplemental Table S2). For example, the pipeline designed a probe set targeting both *S. cerevisiae* and *S. pombe* yeast rRNA by finding regions of identity between each target and then choosing probes corresponding to our selection criteria (Fig. 6B). The yeast probe set was able to deplete the small and large rRNAs to <1% of their input levels (Fig. 6C), similar to the *Drosophila* probe set. In conclusion, we anticipate that Ribo-Pop will prove a versatile and cost-effective tool to enable full transcriptome sequencing for researchers working in many different systems.

## DISCUSSION

We have described a simple, effective, and affordable strategy for ribosomal RNA removal that can be extended to any organism with a sequenced genome. Using only 15 short ∼30mer probes, we were able to reduce rRNA levels greatly and increase the percent of non-rRNA reads from 3% to 73%. Furthermore, by testing individual probes, we elicited information about the importance (or lack thereof) of probe length, *T*_m_, and structural effects. We argue that these insights and the computational probe design pipeline that we have developed will be helpful to any researchers wishing to design their own probes and may even have relevance beyond rRNA removal for other types of RNA pulldown experiments, such as viral RNA purification.

Ribo-Pop has low start-up costs compared to many other noncommerical rRNA subtraction methods. By testing individual probes, we were able to identify features of successful probes and assess how many probes would be needed to obtain the desired results, without resorting to an unnecessarily costly excess of probes and streptavidin matrix. The oligos can be ordered biotinylated for around $1000–$2000, but thereafter used at a relatively low cost per reaction. As described here, oligos can also be ordered unmodified and biotinylated in-house for a start-up cost of around $50 per probe set and the additional cost of the TdT enzyme and ddUTP for a total start-up cost of around $150. The low costs and ease of adding or subtracting probes from the mix will make it simple for researchers to test and optimize custom mixes if needed.

A researcher wishing to deplete as much rRNA as possible may wish to add more probes to target the other contaminants or to tile across the 18S and 28S more densely. However, one should consider whether such a strategy is optimal from a cost-benefit perspective. To do so would more than double the size of the probe set and the cost of the experiment. If the expanded probe set were to decrease rRNA-mapping reads to 5%, then the space available for non-rRNA reads would increase from 73% to 95%, an increase of only 30%. In other words, greatly increasing the target space of the probe set and the cost per experiment is expected to increase the effective sequencing depth only modestly. For this reason, we chose to remain focused on the major contaminants, the processed rRNA transcripts of the small and large cytosolic ribosome. However, our probe design pipeline can accept user parameters to design more densely packed probes or to include more targets if desired.

One potential pitfall of our 15 oligo probe set is that it would not be expected to perform as well on fragmented rRNA. Use of a larger probe set entails a higher cost per reaction but would be predicted to perform better on fragmented samples. Another option for highly fragmented RNA may be an enzymatic depletion method where rRNA is bound by tiling DNA probes and then subjected to RNase H digestion. The RNase H treatment is sometimes preceded by a reverse transcriptase step to extend the DNA/rRNA hybrids and is always followed by a DNAse treatment to remove the DNA probes (Morlan et al. 2012; Fauver et al. 2019). These steps lead to a longer depletion protocol and possibly more opportunities for RNA degradation or nonspecific RNA targeting than Ribo-Pop. Furthermore, RNase H protocols are typically applied to total RNA < 1 µg, presumably due to the cost of reaction scale up for the multiple enzymatic steps. Nevertheless, RNase H methods may be more suitable than pulldown-based methods when dealing with highly fragmented RNA samples.

During preparation of this study, a handful of similar protocols using short probes were published using biotinylated oligos of approximately 50, 40, or 30 nt, followed by streptavidin pulldown (Kim et al. 2019; Kraus et al. 2019; Culviner et al. 2020). Two of these studies were validated with relatively small samples (100 ng) and used 2–3 rounds of depletion on the same sample, resulting in a more laborious protocol that uses many more moles of streptavidin per µg of total RNA than Ribo-Pop (Kim et al. 2019; Kraus et al. 2019). One of these studies, which used 88 40-nt oligos and 60-fold more streptavidin than Ribo-Pop per µg of total RNA (relative to the 1–5 µg experiments, Fig. 3), achieved higher rRNA depletion levels than Ribo-Pop (Kim et al. 2019). We believe that the difference is due to their larger probe set, which includes both denser tiling of the 18S and 28S and inclusion of additional target RNAs. Their expanded probe set and greater decrease in rRNA contamination comes at much higher cost per µg of total RNA that we found unjustified in our experiments. The other study used 90 times more streptavidin despite using only 12 50-nt probes (Kraus et al. 2019). We do not know the reason they used such high molar excess of probes and streptavidin, but we speculate it may be related to the relatively low salt concentration and the inclusion of formamide in their hybridizations, conditions which caused reduced depletion efficiency in our pilot experiments (data not shown). Therefore, we believe that Ribo-Pop is faster and more economical than these related methods.

The most recently published protocol, the DIY method, which used 21 30-nt probes is most similar to ours in probe length, number, and depletion protocol (Culviner et al. 2020). In that study, the authors chose to focus on a design that would be able to target several different bacterial species. Thus, they allowed mismatches between the probe and target and selected probes with a lower *T*_m_ than the Ribo-Pop pipeline. They achieved similar depletion levels to Ribo-Pop, although a direct comparison is difficult because bacteria lack 5.8S and mitochondrial rRNAs. Impressively, they were able to deplete rRNA from highly distant species. The Ribo-Pop probe design pipeline does allow the user to design probe sets targeting multiple species. However, it does not currently support probe design at alignment sites containing mismatches between the target species. Given the prevalence of short stretches of nucleotide identity suitable for probe design and the relatively low cost of creating additional Ribo-Pop probe sets, we do not feel that this feature is required at this time. Users that need to deplete rRNA from a pool of distantly related microbial communities may find the DIY design better suited to their needs, although another option could be to use Ribo-Pop to select high *T*_m_ probes from subsets of more closely related species and then pool them to create a probe set against a very diverse set of species. It is difficult to know whether inclusion of more high *T*_m_ probes or fewer low *T*_m_ probes would lead to better rRNA depletion in this type of experiment. In any case, either of these tools should provide an effective open source option to design rRNA removal probes that target multiple species.

Ribosomal RNA removal reagents should be available to the entire scientific community, not only those that happen to work with the organisms best served by the biotechnology industry. The cost of RNA sequencing has been decreasing rapidly over the last 10 years (Sboner et al. 2011). While previously the cost of the sequencing reaction itself was the main financial barrier to performing these experiments, now with falling sequencing costs, sample preparation costs can become a limiting factor (Sboner et al. 2011). The use of a commercial ribosomal RNA extraction kit can easily more than double the total sample preparation cost. As statistical power and the ability to draw biologically meaningful conclusions grow with the addition of more replicates and more biological conditions (Liu et al. 2014), high sample costs relative to sequencing costs discourage discovery-driven science. Furthermore, the lack of readily available solutions for some model organisms (such as *Drosophila*) and other organisms of ecological interest effectively prevent or severely limit the application of many RNA sequencing protocols in these systems. This discrepancy further widens the gap between often better-funded mammalian work and frequently underfunded work on invertebrate models. We argue that rRNA depletion is the most appropriate approach to informative RNA enrichment for many biological questions, and that “open source” reagents as cost effective and readily available as oligo-dT for poly(A) selection should be made available. We believe that the Ribo-Pop method will help bridge this resource gap.

## MATERIALS AND METHODS

### Probe design for the *Drosophila* rRNA transcripts

We first created an alignment of the *Drosophila* 18S from the three annotated nonpseudogene variants (FBtr0346874, FBtr0346882, FBtr0346878) using MAFFT (Katoh 2002) with the ‐‐auto parameter. A consensus sequence was created in which mismatches and internal gaps in the alignment were replaced by Ns. We input the 18S sequence into the Oligostan.R script (designed for smFISH probe design) (Tsanov et al. 2016) with the default parameters (min length = 26, max length = 32, ΔG_min_ = −36, ΔG_max_ = −28). For the longer 46–52mer probes, we changed the desired probe length to these ranges and set the ΔG_min_ = −56 and ΔG_max_ = −48. We then screened the returned probes to remove probes with five or more consecutive identical nucleotides, four or more consecutive As, or four or more consecutive Cs in the first half of the probe sequence, as previously recommended (Xu et al. 2009; Tsanov et al. 2016). We also removed probes with GC content <40% or >60% (Xu et al. 2009; Tsanov et al. 2016) and probes that overlap with Ns in our consensus sequence of 18S variants. We chose 20 probes of length 26–32 and 5 probes of length 46–52 nt from these sets for our initial tests. We later chose 10 high *T*_m_ probes corresponding to *T*_m_ peaks on the left side of the 18S. The high *T*_m_ probes have otherwise the same length and thermodynamic property ranges (predicted ΔG for homodimer and heterodimer formation) and meet the same sequence composition criteria as the first set of 20 short probes. The predicted *T*_m_ of each probe/target interaction was calculated using Biopython (Cock et al. 2009) with nearest-neighbor values measured for RNA/DNA hybrids (Sugimoto et al. 1995), the DNA concentration set to 250 nM, and salt correction applied (SantaLucia 1998) for 300 mM Na^+^. ΔG estimates were obtained from Primer3 (Untergasser et al. 2012) using a Na^+^ concentration of 300 mM.

After our initial tests we decided to enforce additional probe selection criteria. We limited our selection to probes with a predicted ΔG of hairpin formation >−3 kcal/mol and a predicted ΔG of homodimer formation or heterodimer formation with other probes to >−10 kcal/mol. We also incorporated screening for probes with potential off-target binding using BLAST (Altschul et al. 1990) alignment. We built a custom BLAST database containing both spliced transcripts and introns with 40 nt of flanking sequence, then aligned the reverse complement of our probe candidates to the transcript database. To ensure we detected short regions of homology, we used the blast command “blastn -task blastn-short -dust no -soft_masking false -evalue 50”. We removed any probes that had an alignment bitscore >32, which catches probes with 16 or more contiguous matches to a nontarget transcript. This filter is similar to one used for microarray probe selection (Kane 2000; Wang and Seed 2003).

The five probes targeting the 18S rRNA that were included in the final mix were picked from the initial set of 30 tested probes after checking that they met our additional thermodynamic and specificity criteria. For probes targeting the *Drosophila* 28S rRNA, we made a consensus sequence of FBtr0346876 and FBtr0346885 and selected probes of 26–35 nt that met the same constraints and fell within the top 5% of *T*_m_ for available candidates in a 200 nt moving window. We excluded probes that overlap regions involved in long-range base-pairing: between ES3 and ES6 in the 18S and between helix 22 and helix 88 in the 28S.

### Probe design for additional species with the Ribo-Pop probe design pipeline

To enable streamlined design of additional probe sets, we wrote a pipeline to automate all tasks of probe design. The pipeline builds a consensus sequence for each target, so that multiple transcript variants and/or targets from multiple organisms can be used as input. The consensus sequences are masked (Frith 2011) to prevent selection of probes overlapping repetitive regions. Candidate probes are then created in the user-defined size and *T*_m_ range and filtered to remove probes with undesirable sequence composition or thermodynamic properties, as described in the previous section. The pipeline automates building of the BLAST transcript database and screening of probe candidates for off-target binding. Finally, the pipeline selects candidate probes at *T*_m_ peaks that are as evenly spaced along each target as possible. The pipeline is available at https://github.com/marykthompson/ribopop_probe_design. The pipeline is implemented in Snakemake (Koster and Rahmann 2012).

### Probe biotinylation and purification

Unlabeled oligos were ordered from Sigma Aldrich or Invitrogen with desalting purification and resuspended in probe resuspension buffer (10 mM Tris 8, 0.1 mM EDTA). The long 46–52 nt probes were subjected to in-house PAGE purification before use. Probes were biotinylated with terminal deoxynucleotidyl transferase (TdT, Thermo Fisher) and dideoxy-UTP-biotin (biotin-11-ddUTP, Jena bioscience) as previously described (Gaspar et al. 2017). To biotinylate single probes for testing, we set up 5 µL reactions containing 100 pmol unlabeled oligos, 500 pmol ddUTP-biotin, and 4U TdT. To biotinylate oligo pools, we set up 15 µL reactions containing 1000 pmol mixed unlabeled oligos, 3000 pmol ddUTP-biotin, and 12U TdT. Reactions were incubated overnight at 37°C in a PCR machine and terminated by heat inactivation at 70°C for 10 min. The 5 µL reactions were purified with the ZR-96 Oligo Clean & Concentrator and 15 µL reactions were purified with the Oligo Clean & Concentrator kit (Zymo Research) and eluted with probe resuspension buffer. Probe concentration was determined using duplicate measurements from a NanoDrop ND-1000 instrument using the ssDNA setting and molarity was determined using the A260 value and the nearest-neighbor extinction coefficient estimated from the OligoEvaluator tool (Sigma Aldrich). Biotinylation efficiency was assessed by running an aliquot of the biotinylated oligo alongside an unbiotinylated oligo of the same sequence and 10 bp DNA ladder (Thermo Fisher) on a 20% TBE-Urea PAGE gel (SequaGel, National Diagnostics) and staining with SYBR Gold (Thermo Fisher). Images of the gels were captured with a LI-COR Odyssey Fc.

### *Drosophila* and yeast culture and RNA extraction

*Drosophila melanogaster* wild type strain Oregon-R flies were raised on standard cornmeal-agar medium at 25°C. Three samples of 40 wandering third instar larvae each were collected and RNA was extracted with TRIzol (Allen 2016). *Saccharomyces cerevisiae* strain BY4741 was cultivated in YPAD media (yeast extract, peptone, dextrose [2% w/v] supplemented with adenine hemisulfate). Yeast were diluted to low OD600 and grown overnight at 30°C with rapid agitation, then harvested in mid-log phase at OD 0.5. Each sample was derived from an independent colony. RNA was extracted from cell pellets using the hot acid phenol method. Pellets from 10 mL of culture were resuspended in 200 µL TES (10 mM Tris 7.5, 10 mM EDTA, 0.5% SDS) and 200 µl acidic phenol (Sigma Aldrich P4682) and heated with agitation at 65°C for 10 min in a thermomixer (Eppendorf ThermoMixer C) at 1000 rpm. After 5 min of cooling on ice, then 5 min at room temperature, the sample was mixed vigorously with chloroform and centrifuged 5 min at 16,000*g* to separate the phases. The aqueous phase was then subjected to extraction with 1 volume phenol:chloroform:iaa (Thermo Fisher AM9730, without added buffer) and a final extraction with 1 volume chloroform. The aqueous phase was brought to 300 mM NaOAc with 3M NaOAc, pH 5.2, and 1 volume of isopropanol was added to precipitate the RNA. Prior to rRNA depletion, 50 µg aliquots of RNA were treated with 10U of TURBO DNAse (Thermo Fisher) for 30 min at 37°C, then purified with the RNA Clean & Concentrator kit (Zymo Research) and eluted with water.

### Quantitative PCR

cDNA synthesis was performed with UltraScript Reverse Transcriptase (PCR Biosystems) in 10 µL reactions containing a mix of RNA, random hexamer (5 µM), anchored oligo-dT primer (1 µM, TTTTTTTTTTTTTTTTTTTTVN, Jena Bioscience), buffer, and 100U UltraScript enzyme. Reactions were denatured 5 min at 70°C with RNA and primers alone, then placed on ice for 5 min. The remaining components were then added and the reactions were incubated for 10 min at 25°C, 30 min at 42˚C, followed by 10 min at 85°C. cDNA was amplified with qPCRBIO SyGreen Mix Lo-Rox (PCR Biosystems) according the manufacturer's instructions in 10 µL reactions with a Bio-Rad CFX96 instrument. The qPCR primer sequences, some of which were taken from a previous study (Harzer et al. 2013), are listed in Supplemental Table S3. Standard curves with cDNA dilutions were routinely performed to ensure the amplification efficiency was close to 100% and no RT controls were also assessed to verify the success of DNA removal from the RNA samples. The amplification Ct values were called automatically by Bio-Rad CFX Manager. Fold changes are derived from the ΔΔ*C*_t_ method. Each target *C*_t_ value was first normalized by *Act5c* (ΔCt), followed by normalization by a control sample (either no probe or input RNA, as indicated in the appropriate figure legend) to produce ΔΔCt.

### Single-probe depletion assay

Biotinylated probes were brought to 0.2 µM in probe resuspension buffer (10 mM Tris 8, 0.1 mM EDTA) and used to deplete rRNA from 200 ng of larval total RNA. To minimize the effect of pipetting error on probe efficacy measurements, each replicate uses a different sample of larval RNA and an independent dilution of biotinylated probe. Each probe was hybridized in a 10 µL reaction containing 200 ng total RNA and 0.5 pmol of probe in Ribohyb buffer (2X SSC, 0.01% Tween-20). Reactions were heated to 70°C for 5 min in 1.7 mL tubes in a thermomixer and then placed at room temperature for 10 min before capture with Dynabeads MyOne Streptavidin C1 (Thermo Fisher). To prepare beads for capture, 2 µL beads per sample were washed 3× in MyOne B & W buffer + 0.01% Tween, 2× in Dynabeads buffer A + 0.01% Tween, 2× in Dynabeads buffer B + 0.01% Tween, 3× in Ribohyb buffer and resuspended in a final volume of 10 µL Ribohyb buffer in a nonstick tube (Eppendorf Protein LoBind). The 10 µL hybridization reactions were added to 10 µL of resuspended beads for a binding reaction volume of 20 µL. Samples were then incubated in a thermomixer at 50°C for 10 min at 1000 rpm, then moved to a magnetic rack. The supernatant (rRNA depleted) was taken, the beads were washed with 20 µL of Ribowash buffer (0.4X SSC, 0.01% Tween), and the second supernatant combined with the first supernatant. The supernatant was diluted 1:8 in water and 4 µL of the dilution was used for cDNA synthesis. A no probe control sample, which consists of RNA subjected to hybridization conditions and bead binding in the absence of an added probe, was included in every experiment. In the test of the requirement of the annealing step (Supplemental Fig. S3), the same protocol was followed except that the no annealing samples were added directly to beads that had been preheated to 50°C for 5 min.

### rRNA removal with pooled probes

A mix of 15 oligos targeting the 18S and 28S were biotinylated. The hybridization reaction volume and probe mix amount was scaled to achieve efficient rRNA depletion from different starting amounts of input RNA. For the lower input amounts (0.5 ng and 100 ng), hybridization was performed in 10 µL reactions containing 16 pmol of probe mix and 4U RNasin Plus (Promega). For the 1 µg input samples, hybridization was performed in 10 µL with 40 pmol of probe mix and 4U RNasin Plus. The 5 µg input samples (used for RNA-seq) were hybridized in 40 µL reactions containing 200 pmol of probe mix and 40U RNasin Plus. Reactions were denatured at 70°C for 5 min and then added to prewashed MyOne C1 streptavidin beads with the supernatant removed such that the volume of the binding reaction remained the same as the hybridization reaction (except for the 5 µg samples, where the volume of the binding reaction was increased to 50 µL). Beads were prepared as for the single-probe assay, except Tween was withheld from Dynabeads buffer A. (We observed that Tween forms precipitates in buffer A after storage for >1 wk and thus decided to omit it for further experiments.) After binding, the supernatant was collected, and the beads were washed with 1 volume of Ribowash buffer and the supernatant collected again. The Ribo-Pop depleted supernatant was cleaned up with 1.8X volume of Mag-Bind TotalPure NGS (Omega Bio-tek) beads. After cleanup, Ribo-Pop depleted RNA was subjected to cDNA synthesis and qPCR analysis, where it was compared to a nondepleted input RNA sample from the same source material. 100 ng of *S. cerevisiae* material was tested with the same protocol as the *Drosophila* 100 ng samples.

### RNA sequencing and data analysis

Ribo-Pop rRNA removal was applied to 5 µg of larval RNA as described in the previous section. A synthetic RNA spike-in mix, Lexogen E0 (Lexogen, cat # 025.03) was added to the sample before rRNA removal at a ratio of 0.09 ng per 100 ng total RNA for quality control. After rRNA removal, fractions of both the input RNA and the rRNA-depleted RNA were used to construct total RNA libraries or poly(A)-primed libraries. We used the QuantSeq 3′ mRNA-Seq Library Prep Kit FWD for Illumina (Lexogen, cat # 015.24) to prepare poly(A)-primed libraries. For the input samples, 500 ng of total RNA was used for library construction. For rRNA-depleted samples, the equivalent amount RNA corresponding to 500 ng of total RNA predepletion was used (11 ng). Libraries were amplified with 12 cycles of PCR. We used the NEBNext Ultra II Directional RNA Library Prep Kit (E7765S) to construct the total RNA libraries. We used the equivalent amount of RNA corresponding to 1 µg of total RNA predepletion (22 ng) or a matched amount of nondepleted total RNA. Libraries were amplified for eight PCR cycles. Libraries were loaded onto an Illumina NextSeq 500/550 High Output v2 cartridge and subjected to single-end sequencing on a NextSeq 500 instrument for 85 cycles.

The raw sequencing data was demultiplexed and converted to fastq format on Illumina Basespace. Then adapters were removed from the sequences. For the NEB libraries, the NEB adapter was removed with Cutadapt (Martin 2011). For the QuantSeq libraries, poly(A) and Illumina adapters were trimmed from the reads using BBDuk (Bushnell, BBMap. https://sourceforge.net/projects/bbmap/) with the parameters “k = 13 ktrim = r useshortkmers = t mink=5 qtrim = r trimq = 10 minlength = 20”, as recommended by Lexogen. *Drosophila melanogaster* genomic and transcript sequences were downloaded from Ensembl (release 99, assembly BDGP6.28). A STAR (Dobin et al. 2013) index was built containing the genomic sequence and the SIRV synthetic spike-in gene sequences (Lexogen). A Kallisto (Bray et al. 2016) index was built with the coding, noncoding, and spike-in transcripts, as well as intronic sequences with 30 nt of flanking sequence on either side. Kallisto was used to estimate transcript abundance using the “-l 1 -s 1 –single –fr-stranded” options for the QuantSeq libraries and “-l 1 -s 1 –single –rf-stranded” for the NEB libraries. Reads were also mapped to the genome with STAR. HTSeq (Anders et al. 2015) was used to count unique-mapping reads per gene with “htseq-count” using the STAR alignments and default parameters. Gene-level differential expression analysis was performed with DESeq2 (Love et al. 2014) using the HTSeq quantification.

Gene-level quantification presented in Figure 4 was obtained by summing the quantification of individual transcripts from Kallisto. For biotype analysis, most genes were assigned to the biotype given in the annotation file. An exception was made for pseudogenes derived from rRNA, which were assigned to the rRNA biotype. The coverage of the rRNA locus presented in Figure 4 was obtained using Samtools (Li et al. 2009) and Bedtools (Quinlan and Hall 2010). After sorting the STAR alignments with Samtools, Bedtools was used to calculate coverage using the -bga option to produce a bedgraph file. Comparison of RNA levels and differential expression analysis between depleted and input samples in Figure 5 was done using the HTSeq quantification of the QuantSeq libraries. To search for potential off-target effects of the Ribo-Pop probes, we used BLAST (Altschul et al. 1990) to query the probe target sites against a database containing the longest transcript of each gene using the parameters “blastn -task blastn-short -dust no -soft_masking false -evalue 500”. We also used another local alignment tool, STELLAR (Kehr et al. 2011) to find longer regions of complementarity with more mismatches between both the probes and the targeted rRNA transcripts. To reduce computation time, we searched only the genes for which DESeq calculated a *P*-value for the comparison between input and depleted samples, choosing the longest annotated transcript for each. We used the parameters “-f -e 0.25 -l 26 -n 10000 -s 10000” to search for complementarity to the probes and the parameters “-f -e 0.25 -l 30 -n 10000 -s 10000” to search for complementarity to representative 18S and 28S transcripts (FBtr0346882 and FBtr0346885). Snakemake (Koster and Rahmann 2012) and Conda (Grüning et al. 2018) were used to run the RNA-seq pipeline and manage software dependencies. Downstream analysis and figure construction was completed using open-source scientific computing software, including Numpy, Scipy, Pandas, and Matplotlib, run in Jupyter notebooks (Hunter 2007; McKinney 2010; van der Walt et al. 2011; Kluyver et al. 2016; Virtanen et al. 2020). Illustrations were made in Inkscape. Further details, including the RNA-seq pipeline and details of downstream analysis are available on Github at https://github.com/marykthompson/ribopop_rnaseq. The specific versions of software used are listed in Supplemental Table S3.

## DATA DEPOSITION

RNA sequencing data, including processed files summarizing the gene-level quantification and differential expression analysis, have been deposited in the Gene Expression Omnibus with accession number GSE150332.

## SUPPLEMENTAL MATERIAL

Supplemental material is available for this article.

## Supplementary Material

Supplemental Material
