# Supplementary material for: Ribo-Pop: simple, cost-effective, and widely applicable ribosomal RNA depletion
Source: RNA. 2020 Nov;26(11):1731–42. doi: 10.1261/rna.076562.120 (PMC7566562; doi:10.1261/rna.076562.120)
Supplement: Supplemental Material [file supp_26_11_1731__index.html]

Ribo-Pop: simple, cost-effective, and widely applicable ribosomal RNA depletion — Supplemental Material 

# Ribo-Pop: simple, cost-effective, and widely applicable ribosomal RNA depletion

## Supplemental Material

- Supplemental\_Figures.pdf
- Supplemental\_Protocol.docx
- Supplemental\_Table\_S1\_18S\_candidate\_properties.csv
- Supplemental\_Table\_S2\_probesets.xls
- Supplemental\_Table\_S3\_oligos\_and\_software.xlsx
