# Supplementary material for: Ribo-Pop: simple, cost-effective, and widely applicable ribosomal RNA depletion
Source: RNA. 2020 Nov;26(11):1731–42. doi: 10.1261/rna.076562.120 (PMC7566562; doi:10.1261/rna.076562.120)
Supplement: Supplemental Material [file supp_076562.120_Supplemental_Figures_.pdf]

Supplemental Figures

Ribo-Pop: simple, cost-effective, and widely applicable ribosomal RNA depletion

Mary Kay Thompson, Maria Kiourlappou, and Ilan Davis

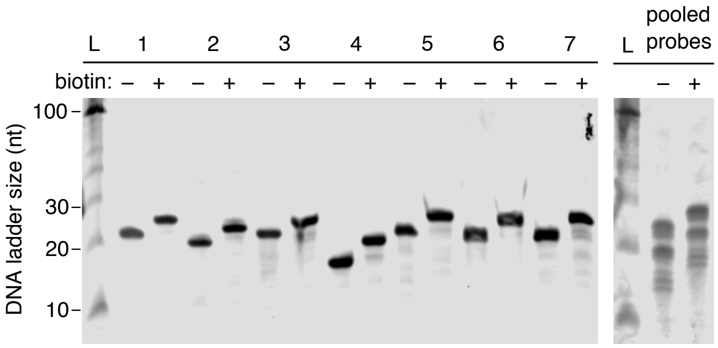

SUPPLEMENTAL FIGURE S1. Enzymatic addition of a single 3′ biotin moiety to unmodified oligos
Unmodified oligos were biotinylated with biotin-ddUTP and terminal deoxynucleotidyl transferase (TdT). Biotinylation efficiency was assessed by Urea PAGE. Successful biotinylation causes an upward shift of the oligo in the gel. In each lane, 1 pmol of oligo was loaded. Left: Biotinylation efficiency assessment for single-probe reactions, a representative gel is shown. Right: Biotinylation efficiency assessment for the pool of 15 oligos. L = DNA ladder.

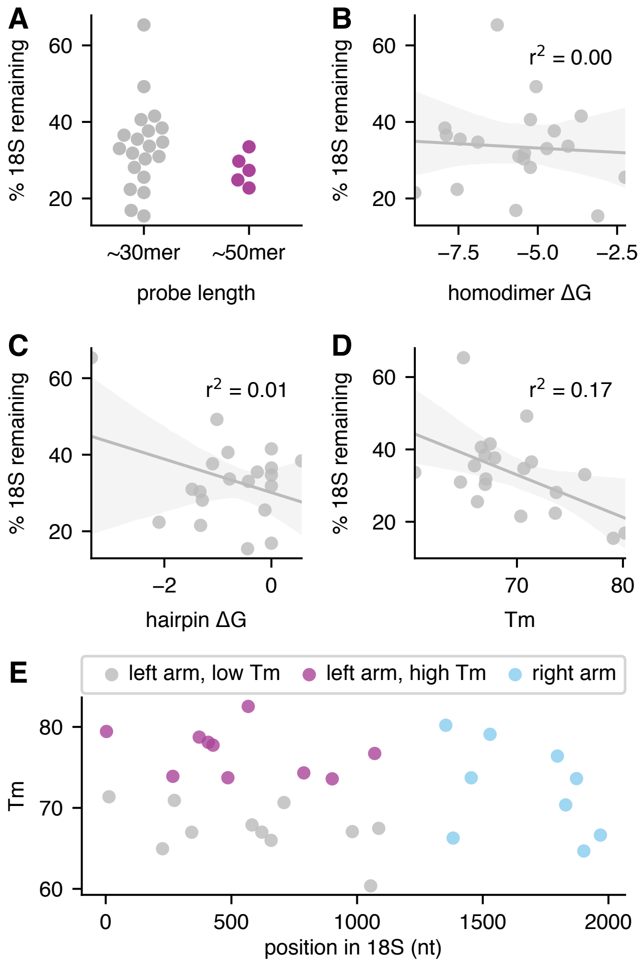

SUPPLEMENTAL FIGURE S2. Correlation of probe properties with performance in the single-probe depletion assay
(A) Comparison of depletion values for short probes (26–32 nt) and longer probes (46–52 nt). Two-sided t-test $p = 0.38$.
(B–D) Depletion values vs. predicted thermodynamic properties of the antisense oligos, including probe self-structure (B & C) and probe/target melting temperature (D). P-values for the Spearman's correlations are 0.99, 0.65, and 0.07 for B–D.
(E) Predicted Tm of the tested probes plotted by target position in the 18S rRNA.

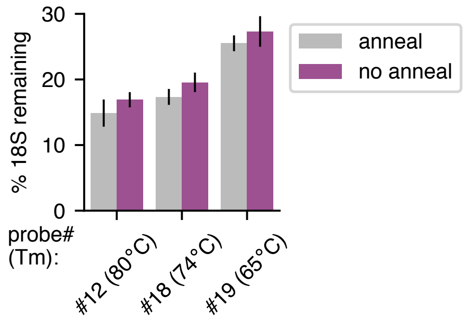

SUPPLEMENTAL FIGURE S3. The effect of removing the annealing step on rRNA depletion
Three different probes were tested for their ability to deplete 18S rRNA from total RNA with or without a 10 minute annealing step after denaturation (allowing the reaction to cool slowly to room temperature before binding to the streptavidin beads). Reactions processed without annealing were added directly to preheated beads after denaturation. The values shown are the averages of three independent experiments using different RNA samples. Error bars are standard deviation. The two-sided paired t-test p-value for annealing vs. no annealing is 0.04.

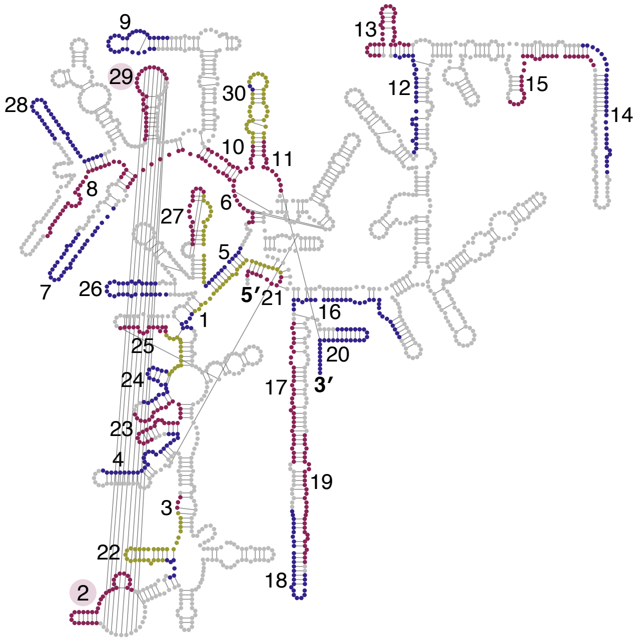

SUPPLEMENTAL FIGURE S4. Probe targeting in heavily structured 18S regions
Target sites of tested probes in the *Drosophila* 18S rRNA, colored as in Fig. 2, but with the detected base pairing interactions shown. Note the interaction between the target sites of probe #2 and probe #29. Some obvious base pairing in helices is absent from the annotated base pairs due to the method of assigning pairing, which was derived from the 3D structure file (Anger et al. 2013; Bernier et al. 2014).

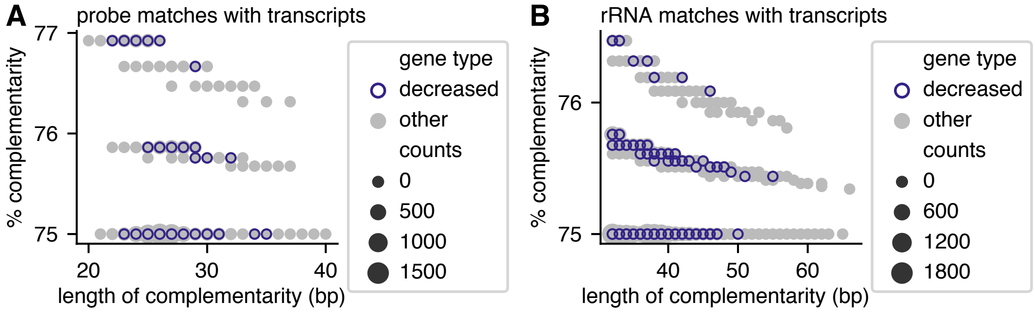

SUPPLEMENTAL FIGURE S5: Complementarity between Ribo-Pop probes or targeted rRNAs and non-targeted transcripts
Regions of complementarity were identified with the local alignment program STELLAR (Kehr et al. 2011). Genes called as decreasing by DESeq2 (Love et al. 2014) (decreased) or those quantified but not called as decreasing (other) are shown.
(A) Probe target sites were aligned with *Drosophila* transcripts to identify potential off-target transcripts. 154/9,151, 1.7%, of reported alignments were with genes which decreased upon Ribo-Pop treatment (compared to 2.8% of genes called as decreasing).
(B) The reverse complements of representative rRNA transcripts for the 18S and 28S were aligned with *Drosophila* transcripts to identify potential hybridization partners of the rRNA transcripts that might be co-depleted by rRNA depletion. 310/20,000, 1.6%, of reported alignments are with genes which decreased upon Ribo-Pop treatment (compared to 2.8% of genes called as decreasing).
